# Capnography Guided Awake Nasal Intubation in a 4 Month Infant with Pierre Robin Syndrome for Cleft Lip Repair-A Better Technique

**Published:** 2009-12

**Authors:** Pramod Patra

**Affiliations:** 1 Consultant, Anesthesiology and Critical Care, Sun Hospital, Cuttack, Orissa

**Keywords:** Pierre Robin Syndrome, Capnography, Difficult airway, Awake nasal intubation

## Abstract

**Summary:**

This four-month-old Pierre Robin child was admitted for cleft lip repair with history of two failed attempts at intubation and subsequent cancellation of surgery. The capnography guided awake nasal intubation was considered as the child's parents were desperate to get the surgery done. A modified cuffless endotracheal tube was used with a capnography sampling tube placed within it. With the capnograph guidance the expiratory gas flow was followed to successfully intubate the child.This technique was found to be very convenient and helpful. The use of this technique in an infant has not been reported so far.

## Introduction

Congenital micrognathia with glossoptosis referred to as Pierre Robin syndrome was described by Pierre Robin in 1923 1. This is a rare syndrome causing air-way obstruction in the neonates and often failure to thrive. When cleft lip is present in a Pierre Robin child, then the airway management becomes a dreaded night-mare to the anaesthesiologist. Apart from its use of monitoring intraoperative ventilation, Capnograph has always been a useful tool in the anaesthesiologist armory and has been used very often to confirm endotracheal intubation in various clinical and prehospital settings 2,3.

Using the Capnogram as a guide to awake blind nasal intubation in an infant has not been reported so far. I report one such unique and novel technique of capnogram guided awake nasal intubation in a case of a four month old Pierre Robin child for cheiloplasty who had two previous failed attempts at intubation under general anaesthesia and subsequent cancellation of surgery.

## Case Report

**Fig 1:**
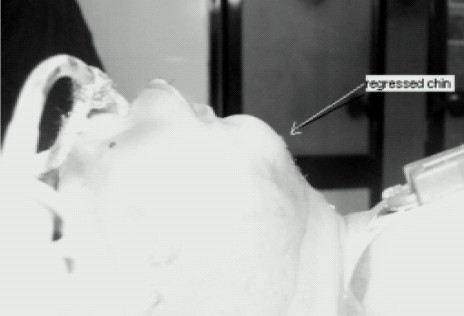
Regressed mandible of Pierre Robin syndrome

**Fig 2:**
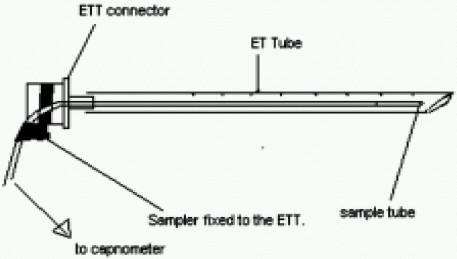
Diagrammatic view of the position of sampling tube inside the ETT

**Fig 3:**
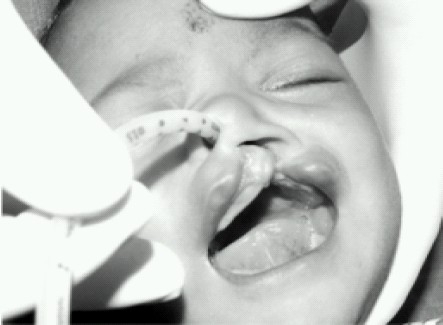
Recording sampling Time

**Fig 4:**
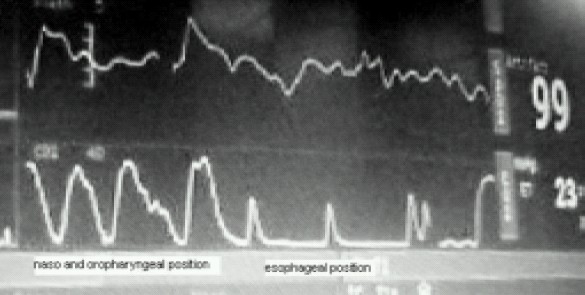
Capnograph showing esophageal and pharyngeal positions

**Fig 5:**
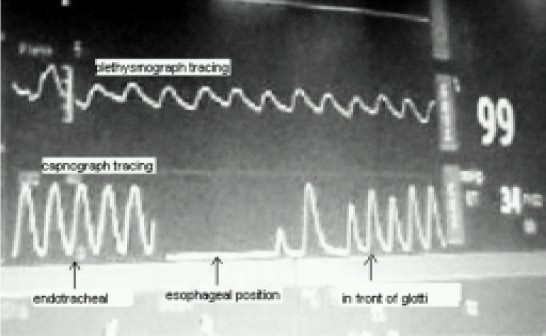
Capnograph showing endotracheal position

A 4 month old male child weighing 5.5 kilograms with cleft lip was admitted for cheiloplasty under general anaesthesia. On preanaesthesia assessment the child was found to have Pierre Robin's syndrome with a grossly regressed mandible, glossoptosis and snoring while sleeping(Fig 1).All routine investigations were within acceptable limits. History from the parents revealed that the child had been posted for the surgery twice before but had to cancel because of failed attempts at intubation. The previous anaesthesiologist had given at least three attempts at two separate occasions after inducing general anaesthesia. Prior discussion with him revealed that mask ventilation was not a problem after inducing GA. Therefore with the surgeons approval It was decided to give another attempt at intubation. Intravenous dexamethasone 2 mg and midazolam 0.3 mg was given IV as premedication. After preoxygenation, induction with intravenous thiopentone 25 mg and succinylcholine10 mg, mask ventilation was done with 100% oxygen and halothane. Laryngoscopy, which was difficult because of the wide cleft on the left side, revealed a Cormak Lehane grade III vision. After two failed attempts with two different sized blades and an attempt with a flexible J tipped CVP guide over wire, it was decided to abandon the surgery. However as the child's’ parents had been trying desperately to get the surgery done, capnography guided awake intubation was considered, while allowing the child to breathe spontaneously. After, confirming from the surgeon that the nasal tube would not interfere with the surgery, a special consent was taken from the parents while allowing the child to awaken fully from the already administered anaesthesia. A 3 mm ID cuffless endotracheal tube was selected through the machine end of which the sampling tube of a side stream capnograph was introduced. The male connector at the tip of the sampling tube needed to be cut so that it could be introduced and be placed just near the beveled end of the ETT(Fig 2). The sampling tube was then fixed to the ETT connector with an adhesive tape, so that the tip does not slip off outside the beveled patient end. To record the Sampling time the patient end of this modified ETT was placed at the patient's nostril during expiration and the time taken for the first upward curve to be seen on the capnograph was recorded as two seconds(Fig 3). The right nostril was sprayed with lidocaine 4 % and a drop of oxymetazoline to provide local anaesthesia and vasoconstriction of the nasal capillaries in order to avoid a messy field. While the child kept spontaneously breathing and crying, the beveled end of the ETT was advanced into the oropharynx with a constant watch on the capnogram .The existence of the expiratory curves on the capnogram while entering the oropharynx suggested that the ETT was in line with the flow of expiratory gases. As the tube was advanced further the EtCO2 tracing disappeared suggesting that the tip entered into the esophagus and was not in the respiratory passage(Fig 4). Withdrawing the tube back till the capnograph reappeared, and with two more similar maneuvers, the endotracheal tube appeared to have passed through the glottis. The instant cough and the capnogram showing regular incursion and excursion with respiration further confirmed endotracheal placement (Fig 5). The EtCO2 reading increased from 18 to 35 mmHg. Following this the sampling tube was withdrawn, the ETT was connected immediately to the breathing circuit and ventilation confirmed manually. The breathing circuit was ready with a side stream capnograph through a second sampling tube in position. Tube position was reconfirmed by bilateral chest elevation and auscultation. The capnogram was regular and further reaffirmed the endotracheal position of the tube. Thereafter atracurium was administered IV and after fixing the tube and packing the throat the patient was handed over to the surgeon to proceed. After adequate reversal from muscle relaxants, extubation was done when the child was fully awake fearing the requirement of reintubation. However the extubation and the subsequent post operative period was uneventful. Surprisingly the snoring as complained by the parents had completely disappeared and there was no stridor after extubation. The child was discharged home after removal of sutures on the 7^th^ day.

## Discussion

Children born with congenital cleft lip and palate are frequently associated with other coexisting complex congenital abnormalities. Pierre Robin syndrome is a very commonly associated congenital anomaly in such cleft children. Markedly regressed mandibles along with a wide cleft in these children usually pose difficulty in obtaining a satisfactory mask fit. In addition, these patients could be difficult to mask ventilate (glossoptosis) and intubate 4. Therefore, use of muscle relaxants is best avoided since airway obstruction is more likely to occur when soft tissues are relaxed 5,6

My case was a challenge as two attempts at intubation had already failed, giving the parents of the child enough worry and anxiety. The surgeon was also equally worried as he found difficulty in explaining the desperate parents that the surgery could be abandoned once again. We had to do something to get the surgery done. We had two options. Either to do it under local anaesthesia along with sedation or retry to intubate the child awake. Local anaesthesia with sedation was risky because, by any chance if there was a need for emergency intubation we would be in serious trouble. So, awake intubation was the only option feasible.

In this child, mask ventilation was not difficult as it had been confirmed in the previous attempts at intubation, and since I had failed in intubating after adequate relaxation, using muscle relaxants again was not considered. I decided to keep the spontaneous efforts intact while going for a blind nasal intubation with capnography guidance. Moreover, the American Society of Anesthesiologists recommend to give priority to the least invasive techniques of airway control and preservation of patients’ spontaneous respiration and consciousness, in all cases of difficult airway.^7^

Awake intubation would perhaps be the safest method of securing the airway. With the child breathing spontaneously, breath sounds and movement of air bubbles in the pharynx has always been a useful guide to intubation. There have been a lot many reported adjuncts which have been successfully used in difficult airway situations, of which the stylets, Bougies and LMAs, are to name a few.There have been reports of the use of the laryngeal mask airway (LMA) in facilitating intubation in pediatric patients with difficult air-ways 8. However in my setup we did not have the smaller size LMA so this option could not be considered.

Although capnograph has been used for rapid confirmation of endotracheal position in both adults and neonates the use of capnography as a guide to oral intubation in adults is currently under study by a clinical 2,3,9,10. It has also been reported by Mentzelopoulos et al that EtCO2 monitoring with a side stream capnograph capable of responding in less than two seconds provided a reliable way to advance the ETT toward the glottis and confirm tracheal intubation 11. Even Jacquelline et al have described capnography as a guide to performing blind oral or nasal intubation in spontaneously breathing adult patients 9.

Bourke and Biehl have gone a step further and developed a special capnograph kit to assist in endotracheal difficult intubation 12.

However in present case I have used the same ETT through which I have passed a capnography sampling tube through the machine end till the patient end. For this the male connector of the sampling tube needs to be cut off so that it can enter into the ETT connector. The advantages being, that the sampling time can be minimized as the gas is directly picked from the beveled end of the ETT when it approximates with the glottis and secondly, once the tube is in the trachea, we just have to pull the sampling tube out and connect to the circuit ready with another sampling tube, thereby saving the time to reoxygenation. One of the main drawbacks of capnography in small children is inaccuracy of the recordings obtained when using non rebreathing circuits. The best method of avoiding artifacts is to sample the expired gases at some point within the endotracheal tube^13^. My technique of sampling directly from the glottis would grossly reduce movement artifacts.

Another important point of discussion in my case is that I have done a nasal intubation while cleft lip surgeries require an oral intubation .This was because a preformed RAE tube would not possibly allow a sampling tube into it. Secondly, it would be difficult to manipulate a RAE tube orally. Thirdly, an awake child would not allow anything to be placed orally and he would possibly use the tongue to displace it. Lastly nasal route is much more stable and guides the tip directly into the glottic opening along its natural passage. However, to ensure that the nasal tube does not interfere with the surgical procedure, the surgeon's prior approval was taken.

To conclude, I would like to emphasize the fact that even in small kids and infants with difficult airway, we can use the capnography as a guide to intubate nasally by keeping the child awake and spontaneously breathing. And a good reliable side stream capnograph would always be another weapon in the anaesthesiologist's armory. I found this technique very helpful and convenient in suspected cases of difficult airways, but its use for routine intubation in otherwise normal airways would be time consuming and cumber-some.
